# Transcriptional analysis in bacteriophage Fc02 of *Pseudomonas aeruginosa* revealed two overlapping genes with exclusion activity

**DOI:** 10.3389/fmicb.2023.1027380

**Published:** 2023-02-03

**Authors:** Irais Ramírez-Sánchez, Marco Magos-Castro, Gabriel Guarneros

**Affiliations:** Departamento de Genética y Biología Molecular, Centro de Investigación y de Estudios Avanzados del Instituto Politécnico Nacional, Mexico City, Mexico

**Keywords:** strand-specific RNA-Seq, overlapping genes, superinfection exclusion, *Pseudomonas aeruginosa*, beetreviruses

## Abstract

Little is known about the gene expression program during the transition from lysogenic to lytic cycles of temperate bacteriophages in *Pseudomonas aeruginosa*. To investigate this issue, we developed a thermo-sensitive repressor mutant in a lysogen and analyzed the phage transcriptional program by strand-specific RNA-Seq before and after thermo-induction. As expected, the repressor gene located on the phage DNA forward strand is transcribed in the lysogen at the permissive temperature of 30°C. Upstream the repressor gene, we noticed the presence of two overlapped ORFs apparently in the same transcript. One ORF is a gene that encodes a protein of 7.9 kDa mediating the exclusion of various super-infecting phages. The other ORF, placed in an alternate reading frame with a possible AUG initiation codon at 25 nucleotide downstream of the AUG of the first gene, is expected to encode a 20.7 kDa polypeptide of yet an unknown function. Upon lifting repression at 40°C, the transcription of an operon which is involved in the lytic cycle is started from a promoter on the reverse phage DNA strand. The first gene in the operon is a homolog of the antirepresor *ner*, a common gene in the lysis–lysogeny regulation region of other phages. Interestingly, the next gene after *ner* is gene *10* that on the reverse strand overlaps the overlapped gene *olg1* on the forward strand. Curiously, gene *10* expression also shows superinfection exclusion. Strand-specific RNA-Seq also has uncovered the transcription succession of gene modules expressed during the phage lytic stage. The conservation of overlapped genes with similar functions may be evolutionarily selected.

## 1. Introduction

During lysogeny, the resident bacteriophage, named prophage, renders the lysogenic cells immune to secondary infection by bacteriophages of the same type as the prophage. This immunity is operated by the repressor, a protein directed by the prophage that turns off its lytic functions as well as those of the incoming homologous phage (Salmon et al., 2000). The prophages also may prevent infection of their hosts by a wider range of other phages different from the resident phage. This event named superinfection exclusion, or simply exclusion, is usually acted upon by proteins encoded in the prophage genome (Hofer et al., 1995; McGrath et al., 2002; Cumby et al., 2012; Ali et al., 2014; Owen et al., 2021). Strain Ps33 of *P. aeruginosa* lysogenic for phage Ps56 is refractory to infection by a collection of hetero-immune test phages that readily infect the non-lysogenic parental strain. It has been suggested that the concerted action of two genes, named *9* and *10*, of phage Ps56 was responsible for the exclusion (Carballo-Ontiveros et al., 2020). It also has been documented that filamentous phages, Pf, inserted into the bacterial chromosome of *P. aeruginosa* confer exclusion by expressing a protein that interacts with PilC, a protein involved in the phage receptor (Schmidt et al., 2022).

It may also occur with interference against superinfection by secondary infecting phages in bacteria already engaged to lysis by previous infection with virulent phages (Shi et al., 2020). In *P. aeruginosa*, cases of genes encoding superinfection exclusion by both temperate and virulent phages have been documented (Bondy-Denomy et al., 2016; Tsao et al., 2018; Schmidt et al., 2022; Xuan et al., 2022).

The phage Fc02 belongs to a family whose archetype is phage B3. The DNA of phage B3 has been sequenced and annotated (Braid et al., 2004). Two possible overlapped promoters were identified in the region that controls the phage lysis–lysogeny responses. Promoter pC2 was identified in the direction of the immunity repressor gene *repc* in the DNA forward strand, and pE, possibly transcribing the antirepresor gene *ner*, in the reverse strand. This promoter configuration is prevalent in other phages (Salmon et al., 2000; Ranquet et al., 2005; Jakhetia and Verma, 2015; Wu et al., 2021). A third possible promoter was also located between ORFs 15 and 16 in the reverse strand of B3 DNA transcribing away from *ner* (Braid et al., 2004). Also, a third possible promoter transcribing in the repressor gene direction was described in the Mu genome (Goosen et al., 1984).

The application of massive techniques of nucleic acids and protein sequencing, as well as of bioinformatics algorithms in biology, has revealed the presence of overlapped genes in genomes more frequently than previously assessed (Wright et al., 2022). The configuration of overlapped open reading frames (ORFs) and their expression signals is variable; they could be overlapped partially or totally in one direction on the same DNA strand or opposite strands and directions (Rogozin et al., 2002; Schlub and Holmes, 2020).

In bacteria and their bacteriophages, numerous cases of extensively overlapped genes have been described (Scherbakov and Garber, 2000; Zehentner et al., 2020; Muñoz-Baena and Poon, 2022). However, frequently the evidence provided is partial, the function of one or both overlapped genes is unknown, or is not clear whether the corresponding mRNA is transcribed and translated into protein. More frequently, the regulation regime of the proteins expressed and their functions are unknown (Kreitmeier et al., 2022). The historic case of overlapped genes in phage φX174 (Sanger et al., 1977) has been updated recently. Data from modified genomes led us to conclude that gene overlapping in the phage genome is essential for the correct expression of proteins and DNA replication (Wright et al., 2020).

In the present study, we have identified a cluster of three overlapped ORFs in the regulatory region that controls the lysogeny and lysis stages of phage Fc02. Two of the ORFs, *olg1* and *olg2*, overlapped in the same direction on the forward DNA strand but in different reading frames. The third ORF, named gene *10*, is overlapped extensively with the *olg1* and *olg2* pair on the DNA reverse strand. Here, we present evidence that *olg1-olg2* are possibly transcribed from a promoter sequence identified right upstream from the genes active during lysogeny. On the opposite strand, gene *10* is part of an operon probably transcribed from a promoter located upstream of the antirepressor gene *ner* early in the lytic stage in the phage development. We show that *olg1* encodes a 7.9-kDa polypeptide that excludes other infecting phages through lysogeny. The olg2 ORF is expected to encode a 20.7 kDa polypeptide without apparent exclusion function.

## 2. Materials and methods

### 2.1. Bacterial strains and phages

Phage Fc02 was isolated from a collection of clinical strains of *P. aeruginosa* (Castañeda-Montes et al., 2018). The strains used in this experiment were *Escherichia coli* DH5α and *P. aeruginosa* PAO1 (Sepúlveda-Robles et al., 2012). Strains were cultured in Luria-Bertani (LB) broth with shaking at 200 rpm at 30°C and 40°C, respectively. The information on the plasmids used in this study is given in Table 1. When required, the following concentrations of antibiotics were used: ampicillin (*E. coli* 100 μg/ml) and gentamicin (*E. coli* 15 μg/ml, *P. aeruginosa* 50 μg/ml).

**TABLE 1:** Strains, plasmids, and phages.

Strains, plasmids, and phages	Genotype or fenotype	References
***E. coli***		
DH5α	F– endA1 glnV44 thi-1 recA1 relA1 gyrA96 deoR nupG purB20 φ80dlacZΔM15 Δ(lacZYA-argF)U169, hsdR17(rK–mK+), λ–	Laboratory collection
***P. aeruginosa***		
PAO1	WildType from our collection	Sepúlveda-Robles et al., 2012
**Plasmids**		
pJET1.2/blunt	Amp*^R^*, rep(pMB1)	Thermo Fisher Scientific
pHERD30T (p*lacZalpha*)	Gm*^R^*, origin pBR322, origin pRO1600, promoter P_BAD_.	Qiu et al., 2008
pSEVA648	Gm*^R^*, origin pRO1600, promoter P_*M*_-*XylS*	Silva-Rocha et al., 2013
pSEVA658-*ssr*	Gm*^R^*, origin RSF1010, P_*M*_-*XylS*, *ssr* gene cloned in the MCS	Aparicio et al., 2016
pSEVA648_*repc*ts	Gm*^R^*, origin pRO1600, promoter P_*M*_-*XylS, repc* ts	This work
pH*olg1* + *olg2*	Gm*^R^*, origin pBR322, origin pRO1600, promoter P_BAD_, *olg1* + *olg2*6His.	This work
pH*olg1*	Gm*^R^*, origin pBR322, origin pRO1600, promoter P_BAD_, *olg1*6His.	This work
pH*olg1*C19	Gm*^R^*, origin pBR322, origin pRO1600, promoter P_BAD_, *olg1*C19.	This work
pH*olg2*	Gm*^R^*, origin pBR322, origin pRO1600, promoter P_BAD_, *olg2*6His tagged.	This work
pHORF10	Gm*^R^*, origin pBR322, origin pRO1600, promoter P_BAD_, ORF10.	This work
pHORF10T13	Gm*^R^*, origin pBR322, origin pRO1600, promoter P_BAD_, ORF10T13.	This work
pH*ORF*10T25	Gm*^R^*, origin pBR322, origin pRO1600, promoter P_BAD_, *ORF*10T25.	This work
**Phages**		
Fc02	Isolated from a clinic lysogen.	Castañeda-Montes et al., 2018
Fc17		
Fc4		
Fc02 *repc* ts	Ts repressor mutant	This work

Amp^R^, ampicilin resistance; Gm^R^, gentamicin resistance.

### 2.2. Random mutagenesis

An exponentially growing culture of PAO1 (Fc02) was mutagenized with nitrosoguanidine (NSG) as described in Vogel et al. (1991) with modifications. Briefly, 7 ml of bacterial culture at an OD_600_ of 0.3 was incubated for 30 min at 30°C with 50 μg/ml of NSG. Cultures were diluted 100-fold and recovered overnight in aeration at 30°C. The bacterial culture was centrifuged at 8,000 rpm for 5 min at 40°C to pellet the cells. The resultant pellet was washed 5 times with 5 ml of LB broth. Finally, the pellet was resuspended with 10 ml of fresh LB broth and incubated with aeration for 3 h at 40°C. The lysogen culture was centrifuged at 10,000 rpm for 10 min. The supernatant was treated with chloroform to remove bacterial remnants, and a virion suspension was carefully recovered (Echeverría-Vega et al., 2019) and plated on PAO1 lawns at 40°C. Clear plaques were isolated and plated on PAO1 lawns at 30°C. The plaques clear at 40°C and turbid at 30°C were isolated, and the lysogen was recovered and isolated from the turbid plaque center at 30°C.

### 2.3. Construction of the *repc* ts lysogen

The PAO1(Fc02 *repc* ts) lysogen was obtained with a modified version of the protocol described in Rice et al. (2009). The point mutation G191A was introduced into the lysogen by homologous recombination. The repressor gene was cloned from the Fc02 ts mutant obtained with NSG, into the vector pJET1.2/blunt, used as a suicide vector. Following the cloning method (ThermoFisher CloneJET PCR Cloning Kit), the pJET suicide vector was purified using the Jena Bioscience Plasmid Mini-Prep Kit and electroporated into PAO1(Fc02) and PAO1(Fc02) harboring pSEVA658-*ssr* (Aparicio et al., 2016). One milliliter of LB medium was added immediately after electroporation and cells were transferred to a 1.5-ml tube and incubated for 4 h at 30°C with shaking at 200 rpm. The culture was centrifuged at 10,000 rpm for 5 min, the supernatant was discarded, and the pellet was washed twice with fresh LB broth. The pellet was resuspended in 1 ml of fresh LB and incubated with shaking at 40°C for 3 h. The culture was centrifuged at 10,000 rpm for 10 min, and the supernatant was collected and treated with chloroform. Finally, the phage in the supernatant was plated on PAO1 loans at 40°C to evaluate the thermo-sensitive phenotype. Independent mutants were purified and saved.

### 2.4. Lysogen growth-curve

Three independent 50-ml cultures of PAO1(Fc02 *repc* ts) were grown on LB broth with 200 rpm shaking at 30°C until OD_600_ was 0.3 and then 2 ml of pre-heated medium was added to rapidly reach 40°C up-shifted temperature. Samples of 1 ml were collected 5, 10, 20, and 40 min after increasing the temperature. The phages at each time point including point 0 were diluted, and the plaques were evaluated in a PAO1 lawn. PFU/ml was calculated and plotted against time.

### 2.5. RNA extraction and sequencing

RNA was extracted from a modified protocol by Blasdel et al. (2018). A total of 50 ml of LB broth was inoculated with 0.5 ml of an overnight culture of PAO1 and PAO1(Fc02 *repc* ts) and incubated with shaking at 200 rpm at 30°C until an optical density at 600 nm (OD_600_) of 0.2–0.3 was reached; the three independent cultures of each condition were collected at 0, 5, 10, 20, and 40 min after thermal shifting to 40°C. Cell suspensions were treated with cold stop solution (95% EtOH, 5% phenol) and placed on ice until samples were pelleted by centrifugation for 20 min at 40°C and 8,000 rpm. After removing the supernatant, the cells were resuspended in a lysis buffer, and the total RNA was isolated with a hot phenol extraction protocol, followed by ethanol precipitation. DnaseI (Thermo Fisher Scientific) treatment was done and hot phenol extraction was repeated to remove any residual DnaseI. The integrity of the RNA was evaluated on a 1% agarose gel, and RNA concentration was determined by NanDrop2000 (Thermo Fisher Scientific). To verify the absence of residual gDNA, we performed PCR on the RNA samples. RNA samples were sent to GENEWIZ (New Jersey, USA) for Strand-Specific RNA sequencing using the Illumina HiSeq platform and 150-bp paired-end reads.

### 2.6. Bioinformatics analysis

For the analysis of the 3D-structures prediction, we used the software I-TASSER (Yang et al., 2015), and the resulting model was compared with lambda CI repressor crystal structure under accession number PDB3J50; alignment of the two structures was done in ChimeraX. For the search of transcriptional regulators, we used BProm, SAPPHIRE, PhagePromoter, Promoter Prediction by Neural Network, FindTerm, and ARNold (Reese, 2001; Naville et al., 2011; Solovyev and Salamov, 2011; Coppens and Lavigne, 2020). The fasta sequence was uploaded in ORF Finder for ORF search (Stothard, 2000). The predicted ORFs were analyzed using BLASTp against non-redundant protein databases (Altschul et al., 1997). Illumina fastq files (see data availability statement below) were used as the input in TRIMMOMATIC (Bolger et al., 2014) and FastQC. For the alignment, we used HISAT2 (Kim et al., 2019) with reference genomes Fc02 (MH719189.1) and PAO1 (NC_002516.2). Coverage plots were created using custom scripts in R. Table counts were created from the counts of the Fc02 and PAO1 genes using Deseq2 software to calculate differentially expressed genes between the various time groups (Love et al., 2014). Genes with a normalized counts fold change (FC) ≥ 1.5 and ≤−1.5 and a *p*-value of ≤1 × 10^–5^ were considered differentially transcribed. The Venn diagrams were obtained using the jvenn viewer (Bardou et al., 2014).

### 2.7. Translatability of genes and exclusion activity evaluation

The gene *repc* was cloned into pSEVA 648 (Silva-Rocha et al., 2013) and the genes *olg*1, *olg2*, and ORF10 were cloned with *Eco*RI and *Hin*dIII into plasmid pHER30T (Qiu et al., 2008) in a 2-step modification, the first to introduce the genes and the second to remove 20 bases from the start of lacZα using QuikChange II Site-Directed Mutagenesis Kit (Agilent) (see primers in Table 2), and finally electroporated into PAO1. The exclusion phenotype of the ORF’s overexpression was evaluated by infection assay with test temperate phages on agar plates supplemented with gentamicin (Carballo-Ontiveros et al., 2020). Protein expression was performed by growing the transformed bacteria in LB medium supplemented with gentamicin to OD_600_ of 0.3 followed by induction with 0.3% of arabinose for 3 h or overnight. The expression of the genes *olg1* and *olg2* was determined by adding a 6 Histidine Tag at the 3′ ends, and protein enrichment and purification were performed as in Bertani et al. (1999) using a Ni^2+^ nitriloacetic acid metal-affinity column according to the manufacturer’s instructions (QIAGEN). Proteins were resolved by Tris-tricine-SDS-PAGE (Schägger, 2006) and stained with Coomassie brilliant blue R-250 after electrophoresis, or, alternatively, proteins were transferred onto a nitrocellulose membrane. Western blot analysis was performed using a polyclonal antibody against the 6Histidine tag (Sigma-Aldrich). After incubation with the second anti-mouse antibody (Invitrogen), the purified protein was detected with SuperSignal™ West Femto (Thermo Fisher Scientific) and scanned with the LI-COR C-DiGit. The insertion *olg1*C19 and the ORF10 substitutions C13T and G25T to generate stop codons on constructs harboring the respective genes were generated using specific primers (Table 2) with QuikChange II Site-Directed Mutagenesis Kit (Agilent).

**TABLE 2:** List of primers used to clone Fc02 ORF’s in pJET1.2/blunt, pHERD30T, and pSEVA648.

Primer	Sequence	Purpose
F_*repc*ts	GAATTCATCGGGGATCGGATC	Clone *repc*
R_*repc*ts	AAGCTTTCATCCTGCTTTCTTACC	
F_*olg1*	GAATTCATGACTACCCCCGATCACCTGGTTCTTGATGCG	Clone *olg1*
R_*olg1* × 6H	AAGCTTGCAGTGATGATGATGATGATGGCTTCCGCCTGCTTGAGC	
F_ORF10	CTTTAAGAAGGAGATATACATACCCATGCCTCGCTTTCAGCCGCCGGT	Clone ORF10
R_ORF10	GTAAAACGACGGCCAGTGCCAAGCTTCTACCCCCGATCACCTGGTTCTTGATG	
F_mut_*olg1*	CTACCCCCGATCACCTGGTTCTTGATG	Edit *olg1*
R_mut_*olg1*	CATCAAGAACCAGGTGATCGGGGGTAG	
F_*olg2*	GATATACATACCCATGCGTCGATGCGCTCGG	Clone *olg2*
R_*olg2*	CCGAGCGCATCGACGCATGGGTATGTATATC	
F_mut_pH30	GAG ATA TAC ATA CCC ATG ACT ACC CCC GAT CAC	Edit pHERD30T
R_mut_pH30	GTGATCGGGGGTAGTCATGGGTATGTATATCTC	
F_mutT13_ORF10	CATGCCTCGCTTTTAGCCGCCGGTCGAG	Edit ORF10
R_mutT13_ORF10	CTCGACCGGCGGCTAAAAGCGAGGCATG	
F_mutT25_ORF10	CTTTCAGCCGCCGGTCTAGCACATCGACCTG	Edit ORF10
R_mutT25_ORF10	CAGGTCGATGTGCTAGACCGGCGGCTGAAAG	

## 3. Results

### 3.1. Generation and characterization of bacteriophage Fc02 thermo-sensitive repressor mutant

**FIGURE 1:**
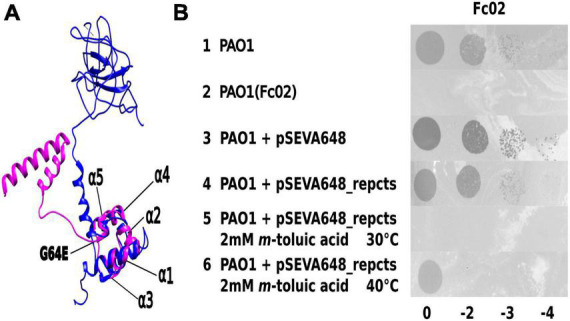
Identification of phage Fc02 immunity repressor gene *rep*c and thermolability of *repc* ts mutant. **(A)** Model of the structural homology of phage lambda CI repressor X-ray crystal resolved structure (Stayrook et al., 2008) (blue) and the I-Tasser-predicted 3D structure of Fc02 ORF12 putative protein RepC (magenta). Notice the N-terminal domains overlapping of the proteins. The *repc* ts mutation G64E was located just beyond the last carboxy-side α-helix of the HTH motif of the putative protein. **(B)** Drop dilutions lysis pattern of phage Fc02 on the indicated strains and temperatures. The phage stock decimal dilutions used in each column are indicated at the bottom of the panel. Notice that Fc02 infects the uninduced transformant (row 4), as it does to wild type strain (row 1), but is unable to infect the *m*-toluic induced transformant at 30°C (row 5) and the PAO1(Fc02) lysogen (row 2).

We approached the study of the transcriptional transition from the lysogeny to the lytic state of Fc02 by creating a thermo-inducible prophage variant (Liu et al., 2013). A culture of the lysogenic strain PAO1(Fc02) was mutagenized with nitrosoguanidine, up-shifted from 30 to 40°C, and the supernatant was plated on a PAO1 lawn on agar plates to select for clear plaques at 40°C that yielded turbid plaques at 30°C, the expected phenotype for the searched mutant. In one candidate, we confirmed the presence of a mutation in the repressor gene by sequence and function. The position of the putative repressor gene in the genome of Fc02 was inferred by informatics-assisted homology with the annotated sequence and lambda repressor (Carballo-Ontiveros et al., 2020). Sequencing of the presumed repressor gene from the isolated candidate showed the unique mutation G64E (Figure 1A and Supplementary Figure 1). We cloned the mutant repressor gene in a suicidal vector to recombine the mutation into a wild-type lysogen PAO1(Fc02) (see 2. Materials and methods). Alignment of an informatic 3-dimensional model of Fc02 repressor protein, RepC, compared with the structure solved for CI lambda repressor showed that the structures overlap closely in the HTH domain (Stayrook et al., 2008). Accordingly, the amino acid substitution in the *repc* ts mutant occurred in the second amino acid beyond the α5 helix of the HTH motif. The thermal sensitivity of the mutated repressor was functionally confirmed by the phenotype of PAO1 bacteria transformed with a construct harboring the mutant repressor gene. This transformant was refractory to infection at 30°C but produced plaques upon infection with a Fc02 phage stock at 40°C (Figure 1B).

### 3.2. Phage Fc02 transcription profile upon thermo-induction of a *P. aeruginosa* lysogen

**FIGURE 2:**
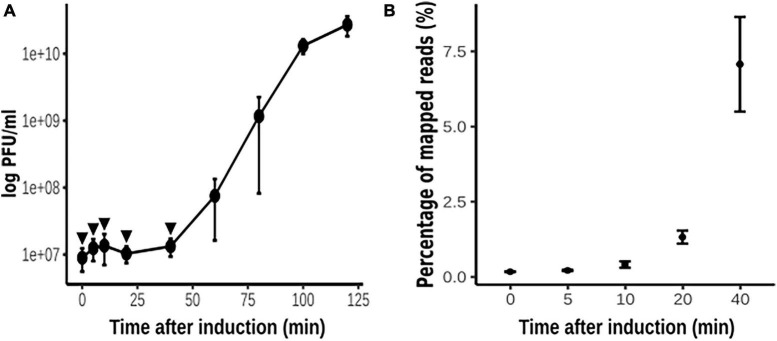
Thermo-induction of lysogen PAO1(Fc02 *repc* ts). **(A)** Time course of the phage growth as measured by plaque forming units (pfu) on a lawn of strain PAO1. Three independent cultures of the lysogen were grown at 30°C to log phase, and at t = 0, each culture was up-shifted and incubated at 40°C for 2 h. Samples were drawn at the indicated times (filled circles) to quantify pfus. **(B)** Samples (arrowheads in **A**) were drawn at the indicated times to be processed for strand-specific RNA-Seq (see Figure 3). Most of the reads were mapped to the bacterial sequences, but the percent of reads that were mapped to the phage genome is plotted against the time of thermo-induction. The average of the pfu/ml and the reads for the three biological replicas at each time were plotted and error bars indicate standard deviation.

To investigate the pattern of phage gene transcription during phage development, we designed an experiment of phage thermo-induction of the lysogen PAO1(Fc02 *repc* ts) (Figure 2A) and evaluate phage RNA at appropriate time intervals. It has been documented that long before the phage progeny appears in the medium, phage genome transcription in the cell has ended (Wang, 2006). This seems to be the case for the induction of Fc02 (Figure 2B). The last point analyzed for the phage DNA transcription was at 40 min after thermo-induction, a few minutes ahead of the time of phage release to the medium and a decrease in the number of viable cells (Supplementary Figure 2).

**FIGURE 3:**
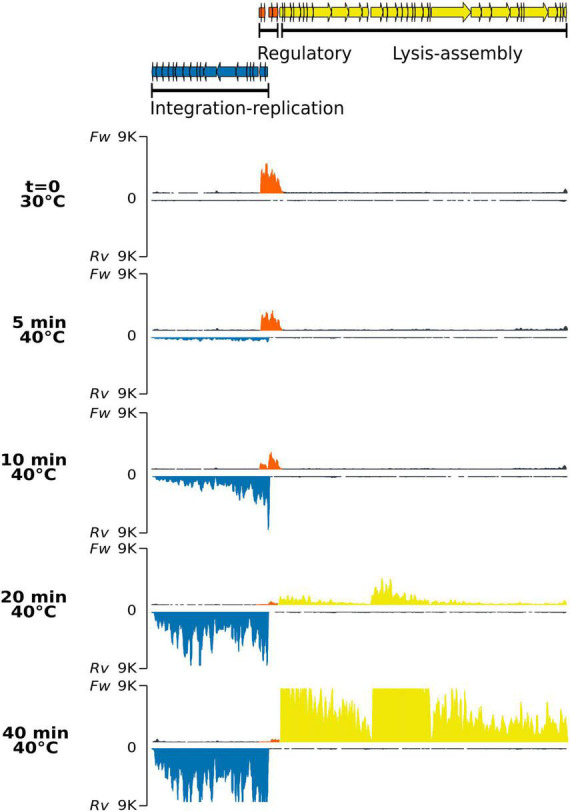
Strand-specific RNA-Seq of the phage genome after thermo-induction of lysogen PAO1(Fc02 *repc* ts). Samples of the lysogen culture grown at 30°C correspond to the uninduced culture (t = 0), and samples after being up-shifted to 40°C were drawn at the indicated times. For each time sample, the upper trace corresponds to RNA reads mapped to the forward strand (*Fw*) and the lower trace, in an inverted scale, to the reverse (*Rv*) strands for each pair. The colors of the read-blocks correspond to the group of genes in the gene map at the top of the figure: orange, genome region transcribed during lysogeny at 30°C; blue, left-arm lytic cycle genes, and yellow, right-arm lytic cycle genes. The histogram for the reads was generated by *ggplot* with 9 K reads maximum set (vertical axis) and map length in nucleotides (horizontal axis).

The wild-type lysogen PAO1(Fc02) growing on a liquid medium at 30°C did not significantly increase the release of infective phage upon shift to 40°C (Supplementary Figure 3). Therefore, no RNA-Seq sample was analyzed for this strain. To investigate the gene transcription program of Fc02 in a lysogen in transition to the lytic mode, a lysogen growing at 30°C was up-shifted to 40°C, and the samples were taken at various times (Figure 3). The profile shows that only the forward strand mapped sequences in the regulation region. The transcripts include the *repc* gene as expected for a lysogen. The transcribed region of about 1,900 nucleotides also includes ORFs in the DNA segments before and after the de *repc* gene. The ORF downstream of the repressor gene is the accessory gene, *e*4 without an assigned function. This gene is not present in all the genomes of beetreviruses (Carballo-Ontiveros et al., 2020). Interestingly, upstream of the repressor gene, two overlapped ORFs were identified on the forward strand of the phage DNA. The ORFs, named *olg1* and *olg2*, appear to be transcribed from a promoter sequence, named pC1, located right upstream *olg*1. Presumably, the *olg1* and *olg2* are encoded in the same mRNA, and therefore might be expressed according to a so far unknown control regime (see section 3.4. “Identification of overlapping genes *olg1* and *olg2*” below) To our knowledge, no such a promoter driving transcription of an overlapped ORFs system had been identified before in the B3-like genome sequences or other Mu-like phages described so far.

Transcription of the *ner*-like gene, mapped to the reverse strand, seems to rapidly ascend by 10 min. Gene *ner* in other phages encodes a protein that turns off *repc* transcription preventing RepC synthesis (Oppenheim et al., 2005; Hulo et al., 2015). Transcription of *ner*-like gene, by itself might antagonize transcription of sequences upstream of the repressor gene. In spite of this transcription reduction, the *repc-e4* mRNA remains in the cell for some time. We do not know whether this transcript was initiated from pC2 *de novo* or if it is a remnant of the RNA initiated at pC1.

At 10 min after the up-shift of a lysogen culture to 40°C, the pE promoter was activated with a burst of transcripts starting at the antirepresor gene *ner* and dwindling distally on the early lytic genes on the reverse strand of the phage DNA. The levels of the pE operon transcripts increased up to a maximum at 40 min, the last time recorded (Figure 3 and Supplementary Figure 4). Most of the ORFs in the pE operon are not assigned to any function except *transposase A*, *transposase B, gemA*, and *mor* homologous genes. In phage Mu, *transposase A* participates in replicative transposition during the phage lytic cycle (Montaño et al., 2012), and *mor*, the last gene in the left operon, is an activator of genes in the middle operon (Mathee and Howe, 1990). Other genes in the left operon correspond to accessory genes in a plasticity genomic region, i.e., that could be present or not in other beetreviruses (Carballo-Ontiveros et al., 2020).

Right downstream to the pair of genes *repc-e*4 on the forward DNA strand are located three clusters of genes sequentially transcribed after thermal induction. The first cluster starts with the holin-endolysin-like genes followed by several DNA packaging genes (Figure 3 and Supplementary Figure 4). The first group of genes is barely transcribed at 20 min when the genes of the pE operon have nearly reached their maximum level of transcription, whereas the second cluster of genes shows incipient transcription. This cluster includes mainly virion structural genes and appears to be limited by a rho-independent transcription terminator (Supplementary Figure 5). The third and first clusters of genes on the forward strand show synchronous levels of transcripts ending at 40 min (Figure 3 and Supplementary Figure 4). Some accessory genes are intercalated in each of the clusters. The gene expression pattern by induction was confirmed by the differential expression analysis (Supplementary Figure 6).

### 3.3. Gene structure of the lysis-lysogeny regulatory region of bacteriophage Fc02

**FIGURE 4:**
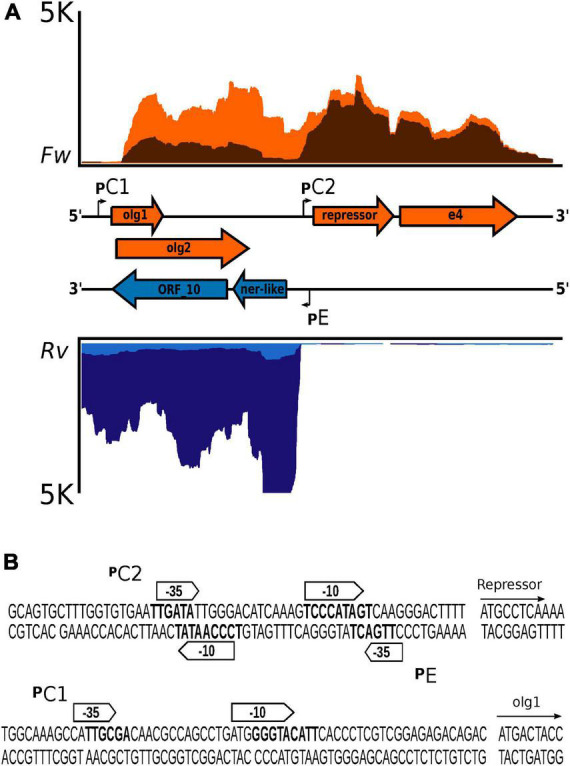
Genetic configuration and promoter sequences identified in the lysis–lysogeny regulation region of bacteriophage Fc02 genome. **(A)** Blow up of the strand-specific RNA-Seq reads mapping in the regulation region (lysogeny-lysis switch) in Figure 3. Graphs in orange represent the reads coverage mapped to the forward strand (*Fw*) at 5 min (light orange) and 10 min after induction at 40°C (dark orange). Graphs in blue, inverted relative to the orange plots, denote the reads coverage of the reverse strand (*Rv*) at 5 min (light blue) and 20 min (dark blue) after induction. In the middle, separating both graphs is presented in a map to the scale of the genes (colored arrows) and the location of the promoter sequences (angled arrows). The genes *olg1* and *olg2* are overlapped in the zero and +1 frames. The *e4* arrow is a putative accessory gene of unknown function. In both coverage graphs, the upper limit was ±5 K reads (vertical axis). The horizontal axis represents the 1,980-nucleotide length of the regulation region. **(B)** Sequence detail of the putative promoters identified using BProm, SAPPHIRE, and Promoter Prediction.

The regulatory region in the Fc02 genome follows the genetic configuration of the B3-like phages of *P. aeruginosa*. In fact, transposable phages of other bacteria share with Fc02 the genetic structure of the regulatory region (Figure 4; Stoddard and Howe, 1989; Fogg et al., 2011; Cazares et al., 2014). Repressor and anti-repressor genes are oriented in opposite directions separated by a non-coding DNA stretch containing the overlapping promoters historically named pRM and pR, necessary for the establishment of repression (Echols and Green, 1971; Spiegelman et al., 1972; Hayes and Slavcev, 2005). In Fc02, the DNA stretch is rather short (106 bp) such that the sequences of the promoters pC2 and pE overlap with some nucleotides (Figure 4B). At 30°C, transcription of the repressor gene appears to occur from the distal pC1 promoter (Figure 4A). However, from the forward transcription profile, it appears that transcription from pC2 is active at 10 min after induction, whereas pC1 activity seems to fade away gradually after induction. The presence of pC1 is rather conserved among the *P. aeruginosa* phages of the B3 family indicating its essential role in the phage lysogenic cycle (Supplementary Figure 7).

### 3.4. Identification of overlapping genes *olg1* and *olg2*

**FIGURE 5:**
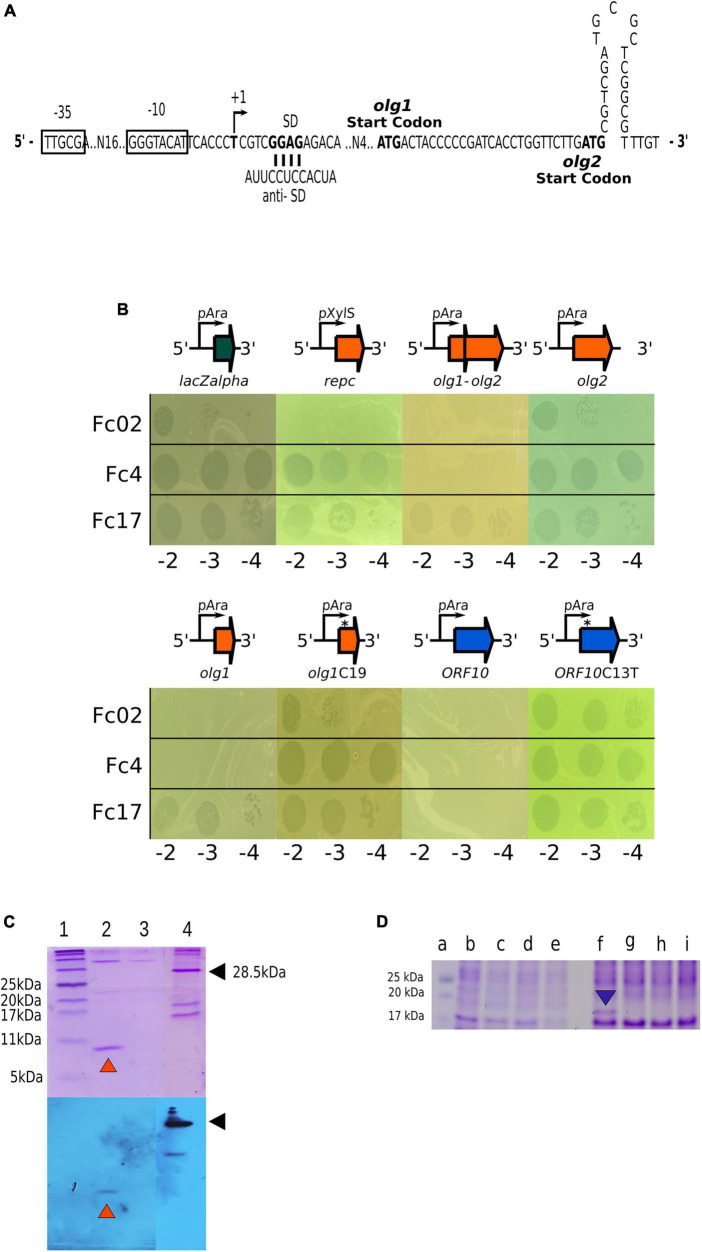
Exclusion activity of Olg1 and ORF10. **(A)** Sequence detail of the overlapping genes promoter pC1 and nucleotide sequence between putative ATG start codons of *olg1* and *olg2*. A possible stem-loop terminator structure right after *olg*2 start is shown. **(B)** Olg1 and ORF10 exclude other infecting phages. Drop assays were carried out with the indicated phage dilutions on induced (arabinose 0.3%) lawns of PAO1 harboring different pHERD30T-based constructs: p*lacZalpha*, empty vector; p*repc*, with phage Fc02 immunity repressor gene; pH*olg1*-*olg2*, with *olg* genes in the phage configuration; pH*olg1*, with *olg*1 only; pH*olg1*C19 with mutant *olg*1 with a cytosine insertion at position 19; pH*olg2*, with *olg*2 moved within a consensus distance from the vector SD sequence; pH*ORF10*, with gene *10* of the reverse DNA strand; and *orf*10C13T that creates a stop codon. **(C)** Protein Olg1 expression from a *olg1-*6His construct in PAO1. Samples were analyzed by Tris-Tricine-SDS-PAGE (16%) followed by Coomassie blue staining (upper panel) or Western immunoblot probed with anti-His antibody (lower panel). Lane 1, BlueRay Prestained Protein Marker (Jena Bioscience); lane 2, Purified Olg1 protein after 3 h L-arabinose (0.3%) induction and Ni^2+^-affinity chromatography, indicated with orange arrowheads; lane 3, Olg1 construct without induction after Ni^2+^-affinity chromatography. Lane 4, a His-tagged protein control, indicated with black arrowheads. **(D)** Expression of ORF*10* variants in PAO1 from wild type and mutant constructs. Samples from overnight cultures induced for ORF10 expression with arabinose were analyzed through Tris-Glicine-SDS-PAGE (16%) followed by Coomassie blue staining. Lane a: Prestained Protein Ladder (MaestroGen AccuRuler RGBPlus); lanes b, c, d and e: soluble fractions; and lanes f, g, h, and i: solubilized pellet fractions. Lanes b and f: pHORF10; lanes c and g: pH*ORF10*C13T; lanes d and h: pH*ORF10C*25T; lanes e and i: pHERD30T empty vector. The blue arrowhead indicates the *ORF10* protein band.

We then asked whether the transcript initiated at pC2 was translated despite being overlapped to gene 10 and partially to the *ner*-like gene in the reverse strand of the regulatory region (Figure 4A). A search using the software ORF Finder revealed the presence of a set of several ORFs; however, we focused our attention on the two overlapped ORFs proximal to the promoter, *olg1* and *olg2*, inscribed within different frames in the forward strand of the regulatory region DNA. The left most ORF, *olg1*, is the closest to promoter pE and has a potential initiation AUG codon properly spaced from a putative SD sequence such that it is expected to be translated. The putative AUG initiation codon for the overlapped *olg2* was located 25 nucleotides downstream in the +1 frame. The final G of this AUG could be the first nucleotide of a stem-loop of a possible rho-independent terminator (Figure 5A). In this work, the interactions of the elements in this complex configuration for the expression of *olg1* and *olg2* were not examined; however, they are tantalizingly interesting to be tackled soon in the future.

As we do not understand how the proposed genes *olg1* and *olg2* are translated from the pC1 transcript and how are they regulated in the lysogen (Figure 5A), we cloned the respective ORFs, in constructs expected to express each of them separately (see 2. Materials and methods and Figure 5B). Based on the proposed exclusion function assigned to the homologous gene *10* of phage Ps56 (Carballo-Ontiveros et al., 2020), translatability and superinfection exclusion of *olg1* and *olg2* was assayed on transformed cells We assessed the exclusion of Fc4, Fc17, and Fc02 phages by cells expressing Olg1 and ORF10 polypeptides from plasmid constructs. Under the used conditions, Olg1 excluded Fc02 and Fc4, and ORF10 excluded all super-infecting phages assayed, but mutant versions of *olg*1 and ORF10 that generate premature stop codons lost their exclusion function in transformed cells. We proved that *olg1* and ORF10 expressed proteins from the respective constructs as detected by SDS-PAGE and Coomassie blue staining. In addition, His-tagged Olg1 protein was identified using Ni-resin enriched preparations and Western blots, and the Gp10 protein band disappeared from the gels by introducing premature stop codons in the ORF 10 sequence (Figures 5C, D). Interestingly, unlike Olg1, Gp10 was not found in the soluble fraction but in the solubilized pellet of the cell lysates (Figure 5D). We were unable to observe the expression of Olg2 or exclusion of the superinfecting phages in cells transformed for *olg*2 constructs, therefore *olg*2 function remains unknown.

## 4. Discussion

In this study, we designed a phage-bacteria system to investigate the prophage transcription regime in the lysogen state and its transition to development lysis stages using a strand-specific RNA-Seq technique. Concurrently, with the induction of the Fc02 *repc* ts prophage, we observed changes in the transcription of the bacterial genes. As expected, the up-regulated genes shared between PAO1 and PAO1(Fc02 *repc* ts) after the first 5 min at 40°C are involved in the heat-shock response (Chan et al., 2016). Many other changes associated with phage development were observed (Supplementary Figure 8 and Supplementary Table 1).

Regarding phage transcription, we found that in the lysogen, the repressor gene apparently is transcribed forward from a distal promoter pC1, but the transcript (ca. 1,700 nucleotides) includes two overlapped upstream genes, *olg*1 in frame + 0 and *olg*2 in frame + 1 on the DNA forward strand (Lèbre and Gascuel, 2017; Muñoz-Baena and Poon, 2022). Two smaller ORFs not studied in this work were also identified. Interestingly, the *olg1-olg2* region completely overlaps gene *10* and part of the 3′-end of the *ner*-like gene on the reverse strand. Remarkably, both *olg1* and gene *10* seem implicated in superinfection exclusion by Fc02 lysogen. Genes *ner* and *10*, in the early lysis left operon on the reverse strand, are transcribed from pE once the lytic response takes over after prophage induction and pC1 is shut off (Figure 4). Although the regulatory switch issue has been studied in other temperate phage models, the expression and function of the ORFs in the pC1 operon require to be investigated in Fc02 to be understood in detail.

In dsDNA viruses, the incidence of overlapping genes is frequent, but these systems are often not well characterized. Either there is no proof that the genes involved are expressed or their function is not clearly known (Schlub and Holmes, 2020; Muñoz-Baena and Poon, 2022). Three-gene overlapping configuration, like the one described here, is a very rare instance but is known to occur in phages (Fiddes and Godson, 1979).

On the forward strand of the phage regulatory region, only a possible initiation AUG in *olg*1 seems to be located at an appropriate distance from a putative SD sequence to be translated (Figure 5). We do not know yet the prophage regulation for *olg*1 and/or *olg2* mRNA translation. For the translation of *olg2*, it is possible that an attenuation mechanism might be involved because, in a sequence model of the transcript, an apparent stem-loop in the mRNA follows the possible initiation AUG of *olg*2 located 25 n downstream *olg*1 initiation codon. Other models of translational control could be frameshifting. The presence of a CCCCCG, a possible slippery sequence followed by a stable secondary RNA structure downstream, could function as frameshift signals (Scherbakov and Garber, 2000). Expression of *olg*1 from an *ad hoc* construct driven by a plasmid promoter shows exclusion properties because transformants expressing Olg1 polypeptide prevent superinfection by a set of test phages (see Figure 5B). Therefore, we propose to rename *olg1* as *sie1* (for superinfection exclusion) following an acronym coined almost 30 years back (Ranade and Poteete, 1993; Hofer et al., 1995). A BLASTp search of *olg*1 showed that most of its predicted amino acid sequence matches a putative conserved domain present in a DEDDh exonuclease family, which could be associated with the exclusion activity at the DNA level.

The reverse DNA strand, opposite to the *olg1-olg2* forward strand, contains gene *10* in beetreviruses (Figure 4). In phage Ps56, akin to Fc02, it has been reported that the gene *10* homolog provides exclusion properties to the lysogenic cells (Carballo-Ontiveros et al., 2020). Remarkably, the expression of Fc02 ORF10 in PAO1 also generates exclusion activity against temperate phages. However, the exclusion mechanisms for the two genes might be different; gene *10* of phage Ps56 seems to exclude by preventing injection of the superinfecting phage DNA (Carballo-Ontiveros et al., 2020). This may explain why ORF10 excludes the hetero-immune temperate phage Fc17 but Olg1 does not, and why Olg1 is found in the soluble fraction of the cell lysates whereas Gp10 is in the insoluble fraction (Figure 5B).

The conservation of overlapped genes expressing proteins with similar functions, in one direction throughout lysogeny and the opposite direction during lytic development, summons us to speculate about the origin and evolution of this novel module of forward and reverse iso-functional genomic DNA. Whether or not they exclude superinfecting phages at different steps of development remains to be explored.

## Data availability statement

The data presented in this study are deposited in the SRA database repository, accession number: PRJNA872573.

## Author contributions

IR-S and GG: design of the study, data analysis and interpretation, drafting the article, and critical revision of the article. IR-S and MM-C: data collection and experiments. All authors contributed to the final approval of the version to be published.
